# Physiological evidence for diversification of IFNα- and IFNβ-mediated response programs in different autoimmune diseases

**DOI:** 10.1186/s13075-016-0946-9

**Published:** 2016-02-17

**Authors:** Tamarah D. de Jong, Saskia Vosslamber, Elise Mantel, Sander de Ridder, John G. Wesseling, Tineke C. T. M. van der Pouw Kraan, Cyra Leurs, Harald Hegen, Florian Deisenhammer, Joep Killestein, Ingrid E. Lundberg, Jiri Vencovsky, Mike T. Nurmohamed, Dirkjan van Schaardenburg, Irene E. M. Bultink, Alexandre E. Voskuyl, D. Michiel Pegtel, Conny J. van der Laken, Johannes W. J. Bijlsma, Cornelis L. Verweij

**Affiliations:** Department of Pathology, VU University Medical Center, Amsterdam, The Netherlands; Department of Molecular Cell Biology and Immunology, VU University Medical Center, Amsterdam, The Netherlands; Department of Neurology, VU University Medical Center, Amsterdam, The Netherlands; Department of Neurology, Innsbruck Medical University, Innsbruck, Austria; Rheumatology Unit, Department of Medicine, Karolinska University Hospital, Solna, Karolinska Institutet, Stockholm, Sweden; Institute of Rheumatology, Prague, Czech Republic; Department of Rheumatology, Amsterdam Rheumatology and Immunology Center, Reade, Amsterdam, The Netherlands; Department of Rheumatology, Amsterdam Rheumatology and Immunology Center, VUmc, Amsterdam, The Netherlands

**Keywords:** Type I interferons, Gene expression profiling, Autoimmune diseases, Rheumatic diseases, Multiple sclerosis

## Abstract

**Background:**

Activation of the type I interferon (IFN) response program is described for several autoimmune diseases, including systemic lupus erythematosus (SLE), multiple sclerosis (MS), myositis (IIM) and rheumatoid arthritis (RA). While IFNα contributes to SLE pathology, IFNβ therapy is often beneficial in MS, implying different immunoregulatory roles for these IFNs. This study was aimed to investigate potential diversification of IFNα-and IFNβ-mediated response programs in autoimmune diseases.

**Methods:**

Peripheral blood gene expression of 23 prototypical type I IFN response genes (IRGs) was determined in 54 healthy controls (HCs), 69 SLE (47 test, 22 validation), 149 IFNβ-treated MS (71 test, 78 validation), 160 untreated MS, 78 IIM and 76 RA patients. Patients with a type I IFN signature were selected for analysis.

**Results:**

We identified IFNα- and IFNβ-specific response programs (GC-A and GC-B, respectively) in SLE and IFNβ-treated MS patients. Concordantly, the GC-A/GC-B log-ratio was positive for all SLE patients and negative for virtually all IFNβ-treated MS patients, which was confirmed in additional cohorts. Applying this information to other autoimmune diseases, IIM patients displayed positive GC-A/GC-B log-ratios, indicating predominant IFNα activity. The GC-A/GC-B log-ratio in RA was lower and approached zero in part of the patients, implying relative importance of both clusters. Remarkably, GC-A/GC-B log-ratios appeared most heterogeneous in untreated MS; half of the patients displayed GC-A dominance, whereas others showed GC-B dominance or log-ratios near zero.

**Conclusions:**

Our findings show diversification of the type I IFN response in autoimmune diseases, suggesting different pathogenic roles of the type I IFNs.

**Electronic supplementary material:**

The online version of this article (doi:10.1186/s13075-016-0946-9) contains supplementary material, which is available to authorized users.

## Background

Type I interferons (IFNs) comprise a large family of cytokines with antiviral, immunomodulatory and anti-proliferative activities. The type I IFN family consists of 17 closely related members, including 13 IFNα subtypes and 4 unique members, i.e., IFNβ, IFNε, IFNκ and IFNω, of which IFNα and IFNβ are most commonly expressed and well-characterized.

Type I IFNs achieve their biological effects by binding to multi-subunit receptors, IFNAR1 and IFNAR2, on the cell surface. This leads to receptor dimerization and activation of the JAK-STAT pathway, a complex cascade of intracellular secondary messengers that emerge in transcriptional activation of genes containing IFN-stimulated response elements (ISRE) and/or IFN gamma-activated sequences (GAS) [1–4]. Upregulation of type I IFN response genes (IRGs) is referred to as a type I IFN signature and is a reflection of type I IFN bioactivity.

Initially, type I IFNs were defined by their antiviral effects and as a consequence, they were used for the treatment of chronic viral infections such as hepatitis B and hepatitis C [5]. The antiviral activity involves suppression of viral replication, induction of apoptosis in virally infected cells, stimulation of T cell and B cell responses, natural killer cell-mediated and CD8+ T cell-mediated cytotoxicity and activation of dendritic cells [6].

Increasing insight in the activities of type I IFNs has revealed their role as pleiotropic cytokines with a critical role in modulating immune responses. Several observations indicate involvement of type I IFNs and the presence of a type I IFN signature in autoimmune diseases, including systemic lupus erythematosus (SLE), Sjögren’s syndrome, systemic sclerosis, multiple sclerosis (MS), idiopathic inflammatory myopathies (IIM) and rheumatoid arthritis (RA) [7–9]. Compelling evidence from studies in SLE demonstrates that IFNα in particular is directly implicated in the pathogenesis of SLE [10, 11]. SLE is characterized by the presence of autoantibodies to nucleic acid and associated proteins, which are able to induce IFNα protein [12]. Serum levels of IFNα are increased in SLE and associated with disease severity and organ involvement [13, 14]. In support of a pathogenic role of IFNα in SLE was the observation that virally infected people and cancer patients treated with IFNα sometimes produce anti-nuclear antibodies and occasionally develop SLE-like symptoms [15, 16]. The mechanisms by which IFNα may contribute to autoimmunity are the induction of autoreactive lymphocytes, enhancement of long-term antibody responses and priming of myeloid cells.

In contrast to the pathogenic effects of prolonged IFNα signaling in SLE, IFNβ administration has notable therapeutic effects in MS, an autoimmune disease of the central nervous system characterized by progressive neurological dysfunction due to demyelination and axonal damage [17]. In patients with MS, treatment with IFNβ reduces clinical relapses and brain disease activity, and slows down progression of disability [18]. The anti-inflammatory and tissue-protective mechanism of IFNβ likely involves anti-proliferative and pro-apoptotic effects, as well as induction of anti-inflammatory mediators such as IL-10, IL-1R antagonist and soluble TNF receptors and reduction of pro-inflammatory mediators such as IL-1, IL-6 and TNFα [19].

From the above, the question emerges why type I IFNs can be pathogenic in SLE but therapeutic in MS. It is tempting to speculate that despite mechanistic similarities, IFNα and IFNβ have distinct roles in immune regulation that confer these opposing effects. Comparison of the primary amino acid sequences reveals that IFNα differs from IFNβ by approximately 70 % [20]. Receptor binding studies demonstrate that IFNα and IFNβ interact with their receptors in a different manner, suggesting that IFNα and IFNβ activate the IFNAR1/IFNAR2-mediated signal transduction pathway in a slightly different way [21–23]. Accordingly, in vitro studies reveal that IFNβ appeared to be more potent at inhibiting cell proliferation and inducing apoptosis than IFNα [24]. However, it is as yet unknown whether there are differences in the downstream gene activation program of IFNα- and IFNβ-induced IFN signatures in vivo.

In the present study, we used transcript profiling to compare the IFN signature gene components regulated by IFNα in SLE patients to those of MS patients who were treated with IFNβ. Moreover, we exploited our findings to delineate the nature of the type I IFN signature in IIM, RA and patients with MS who were IFNβ-naïve.

## Methods

### Patient recruitment

SLE patients (n = 47) and RA patients (n = 76) were recruited at the Amsterdam Rheumatology and immunology Center, Amsterdam, The Netherlands. Patients with MS were recruited from the NABINMS study, a prospective European multicenter study that was previously described [25]. For the untreated MS cohort, blood samples collected before start of IFNβ therapy were used (n = 160); for the IFNβ-treated MS cohort, we used blood samples drawn after 3 months of IFNβ therapy (n = 71). IIM patients (n = 78) were recruited at the Rheumatology Unit at Karolinska University Hospital, Stockholm, Sweden or at the Institute of Rheumatology, Prague, Czech Republic, and fulfilled the diagnostic criteria for definite or probable polymyositis (n = 32), dermatomyositis (n = 40) or sporadic inclusion body myositis (n = 5). Healthy controls (HC, n = 54) were recruited at the VU University medical center, Amsterdam. This study was approved by the medical ethical committees of the VU medical center, the Slotervaartziekenhuis and Reade in Amsterdam, The Netherlands, Karolinska Hospital in Stockholm, Sweden, the Institute of Rheumatology in Prague, Czech Republic and the centers participating in the NABINMS study [25], and informed consent was obtained from all donors. Demographic data, clinical information and medication use of the patients at the time of blood sampling are shown in Table 1.

**Table 1:** Patient characteristics for the complete cohorts or the IFN^high^ selection

		SLE	IFNβ-treated MS^b^	Untreated MS	IIM	RA	Healthy controls
Total amount	All	47	71	160	78	76	54
	IFN^high^	30	63	12	26	10	
Age in years, mean (SD)	All	44 (14)	34 (8)	36 (10)	56 (14)	54 (13)	35 (10)
	IFN^high^	42 (13)	35 (8)	34 (9)	55 (17)	52 (16)	
Female, %	All	85	73	67	62	79	53
	IFN^high^	93	72	83	69	89	
Disease activity, mean (SD)^a^	All	4 (5)	n.a	n.a.	n.a.	4.8 (1.4)^c^	n.a.
	IFN^high^	5 (5)				5.3 (1.6)^d^	
Current prednisolone use, %	All	50	n.a.	n.a.	70	17	n.a.
	IFN^high^	57	n.a.	n.a.	60	22	
Current use of other immunomodulatory drugs, %	All	63	n.a.	n.a.	60	24	n.a.
	IFN^high^	67	n.a.	n.a.	56	33	

^a^Disease activity scores: for systemic lupus erythematosus (SLE), the Systemic Lupus Erythematosus Disease Activity Index; for rheumatoid arthritis, the Disease Activity Score in 28 joints. ^b^Patients with a type I interferon (IFN) signature (*IFN* ^*high*^) (see Fig. 1) before start of therapy were not included in the IFN^high^ selection of this cohort. ^c^data missing from 5 patients. ^d^data missing from 1 patient All refers to the complete cohort. *MS* multiple sclerosis, *IIM* idiopathic inflammatory myopathies, *SD* standard deviation, *n.a.* not applicable

### Blood sampling and RNA isolation

From the donors in the SLE, IIM, RA and HC cohorts, 2.5 ml blood was drawn in PAXgene tubes (PreAnalytix, GmbH, Hombrechtikon, Switzerland) and stored at −20 °C. After overnight thawing at room temperature total RNA was isolated according to the manufacturer’s instructions (PAXgene Blood RNA kit). Total RNA concentration was measured using the Nanodrop spectrophotometer (Nanodrop Technologies, Wilmington, DE, USA). From the donors in the untreated MS cohort and the IFNβ-treated MS cohorts, blood was collected in a Tempus tube (Applied Biosystems, Waltham, MA, USA), and processed as described before [25].

### Reverse transcription and pre-amplification of cDNA

RNA (0.5 μg) was reverse-transcribed into cDNA using a Revertaid H-minus cDNA synthesis kit (MBI Fermentas, Waltham, MA, USA). A single aliquot of each cDNA sample, equivalent to 12.5 ng RNA, was first subjected to 14 cycles of specific target amplification using a 0.2X mixture of all Taqman Gene Expression assays in combination with the Taqman PreAmp Master Mix (Applied Biosystems, Foster City, CA, USA). Following pre-amplification, the samples were diluted 1:5 (v/v) in Tris-EDTA buffer, pH 8.0.

### Multiplex Real-Time PCR

Custom-designed TaqMan®assays for each gene were supplied by Applied Biosystems. Quantitative PCR (qPCR) analysis was performed at ServiceXS (ServiceXS B.V., Leiden, The Netherlands) using the 96.96 BioMark™ Dynamic Array for Real-Time PCR (Fluidigm Corporation, San Francisco, CA, USA), according to the manufacturer’s instructions. Thermal cycling and real-time imaging of the BioMark array was done on the BioMark instrument, and cycle threshold (C_T_) values were extracted using the BioMark Real-Time PCR analysis software. Relative quantities were calculated using the standard curve method, using glyceraldehyde-3-phosphate dehydrogenase (*GAPDH*) as a housekeeping gene. Expression levels were log_2_-transformed.

### Calculation of the type I IFN score and selection for initial analyses

Based on whole genome expression data available in the literature, 23 interferon response genes (IRGs) (see Additional file 1: Table S1) were selected that are reflective of a communal type I IFN signature between autoimmune diseases. All 23 genes are described to be upregulated compared to healthy controls in one or more of the autoimmune diseases SLE, MS, IIM and/or RA [9, 13, 26–30]. To control for inter-experimental variation, expression levels of each gene were calculated relative to the median expression of the gene in healthy controls. Because all IRGs were strongly correlated (Pearson *r* >0.7 for 90 % of the combinations, *p* <0.0005), we calculated an IFN score by averaging the expression levels of all IRGs per sample.

**Fig. 1:**
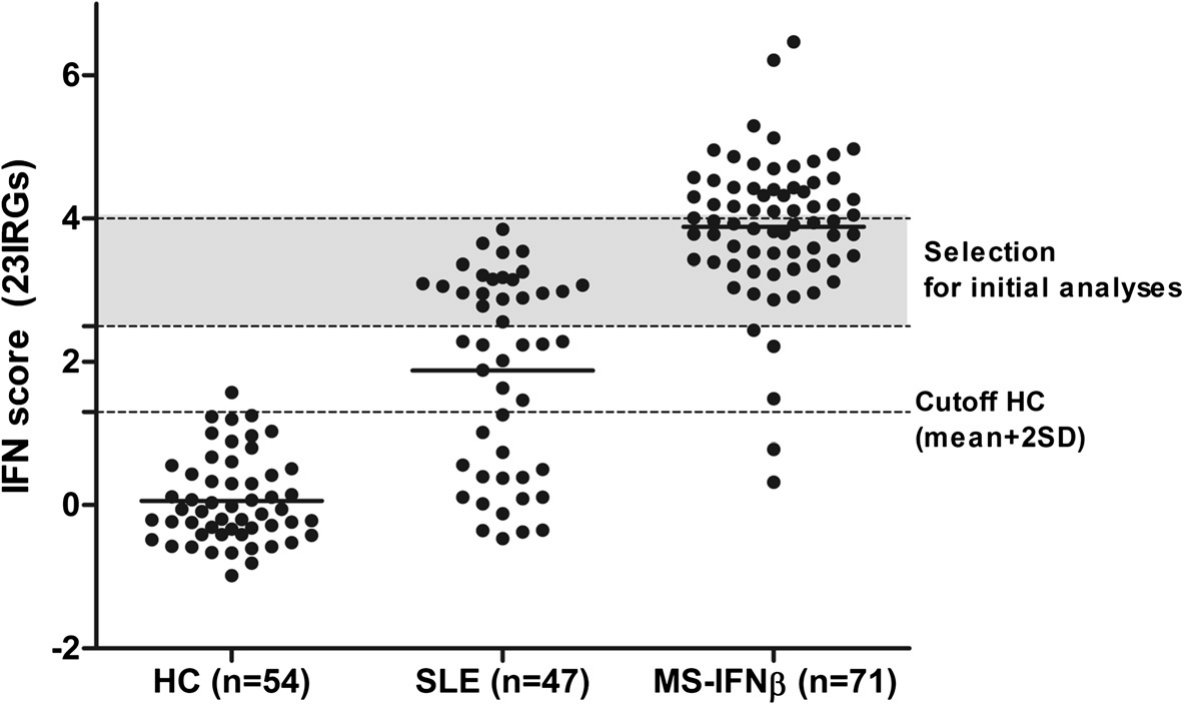
Interferon (*IFN*) score in systemic lupus erythematosus (*SLE*) patients and multiple sclerosis (MS) patients who received 3 months of IFNβ treatment. Average expression levels of 23 interferon response genes (*IRG*s) show a comparable range for the majority of SLE patients and IFNβ-treated MS patients. SLE and IFNβ-treated MS patients with an IFN score between 2.5 and 4.0 (*gray area*) were selected for the initial comparison of the composition of the IFN signature. Patients with a type I interferon signature and an IFN score above 4.0 or below 2.5 were used as an additional cohort. *HC* healthy controls

Presence of a type I IFN signature (referred to as IFN^high^) was defined as an IFN score above mean + 2*SD in HCs (1.3). To exclude the possibility that observed qualitative differences are actually due to quantitative differences, we selected IFN^high^ patients within a comparable range of IFN score, between 2.5 and 4.0, for initial analysis (Fig. 1). The remaining IFN^high^ patients were used as an additional cohort to verify our initial findings.

### Statistical analysis

Cluster analysis was used for categorization of IRGs with respect to their relative expression between diseases [31]. TreeView was used to visualize the clustering of genes (Eisen Lab, Berkeley, CA, USA). Comparison of IRG expression between SLE and IFNβ-treated MS patients was performed using the unpaired *t* test, with multiple testing correction using the Benjamini-Hochberg method. Comparison of IFN scores between SLE and MS-IFNβ was performed using the unpaired *t* test and comparison of cluster-specific IFN scores within patients was performed using the paired *t* test. *P* values <0.05 were considered significant.

## Results

### Differential expression of IRGs in SLE versus IFNβ-treated MS patients

In order to explore in vivo differences in the composition of type I IFN signatures in autoimmune diseases, we studied IRG expression profiles of a prototype IFNα-driven disease, i.e., SLE, and those of MS patients who were treated with IFNβ for 3 months. As described above, only patients with an IFN signature (referred to as IFN^high^) were included for further analysis. To ensure that the observed IFN signature was specifically induced by the IFNβ treatment, MS patients with an IFN signature before start of IFNβ treatment were excluded from analysis. For initial analysis, we used data from patients with comparable levels of IFN score, between 2.5 and 4.0, as described above.

**Fig. 2:**
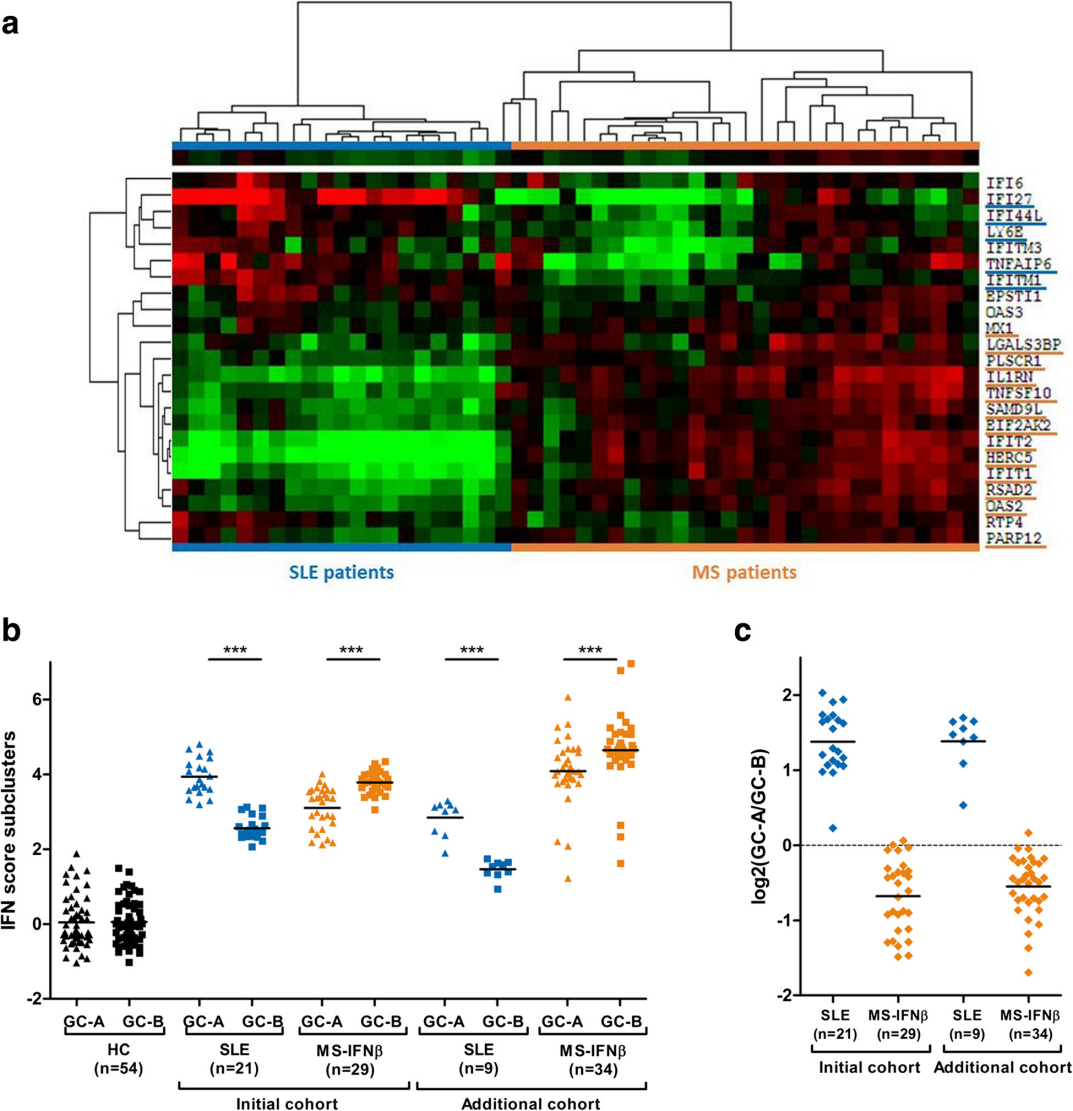
Comparison of gene clusters in systemic lupus erythematosus (*SLE*) and interferon (*IFN*)β-treated multiple sclerosis (*MS*) patients. **a** Unsupervised cluster analysis of SLE patients with a type I interferon signature and IFNβ-treated MS patients. Patient groups were fully separated based on their expression profiles of 23 interferon response genes. Separation is based on differential expression of two major gene clusters. Significantly different genes comprising GC-A (*blue*) and GC-B (*orange*) are *underlined*. **b** GC-A and GC-B scores were compared in SLE and IFNβ-treated MS patients in the initial and additional cohort. In both cohorts, the GC-A score is higher than the GC-B score in SLE patients, whereas the opposite is true for IFNβ-treated MS patients. **c** The log-ratio of GC-A and GC-B scores was compared in SLE and IFNβ-treated MS patients from the initial and additional cohort. In all SLE patients, the ratio is above zero, indicating GC-A > GC-B. In virtually all IFNβ-treated MS patients, GC-A/GC-B ratio is below zero, indicating GC-B > GC-A. *HC* healthy controls

To compare the IFN signature gene components regulated by IFNα in SLE to those of IFNβ-treated MS patients, unsupervised cluster analysis was performed (Fig. 2a). Strikingly, the analysis revealed perfect separation of SLE patients and IFNβ-treated MS patients based on two IRG clusters. From the upper cluster, 5 out of 7 IRGs (GC-A) were significantly upregulated in SLE patients compared to MS-IFNβ, whereas 13 out of 16 genes (GC-B) from the lower cluster were significantly upregulated in the IFNβ-treated MS patients compared to SLE (Fig. 2a and Additional file 1: Table S2). GC-A and a GC-B scores were calculated by averaging expression values of these 5 and 13 genes, respectively. As shown in Fig. 2b, the GC-A score was significantly higher than GC-B in SLE (*p* <0.001) whereas the GC-B score was significantly higher than GC-A in IFNβ-treated MS (*p* <0.001). Analysis of IFN^high^ patients with IFN scores lower than 2.5 or higher than 4.0 confirmed these findings (additional cohort, Fig. 2c). To gain insight into the relative importance of each gene cluster per patient, the GC-A/GC-B ratio was calculated. As this ratio is based on log_2_-values, a ratio above zero means a higher GC-A score compared to GC-B, whereas a ratio below zero means that the GC-B score is higher than the GC-A score. Comparison of these GC-A/GC-B log-ratios revealed that SLE and patients with MS from both cohorts could be completely separated based on these ratios (Fig. 2c).

### Validation in public microarray datasets

**Fig. 3:**
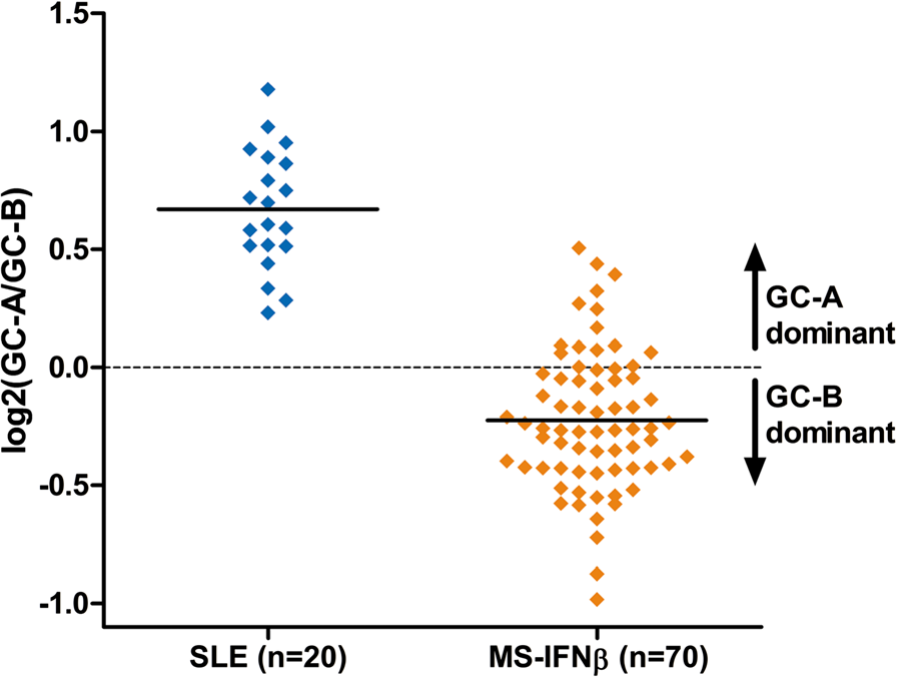
Validation of our findings in publicly available microarray data. The ratio between GC-A and GC-B was calculated for independent validation cohorts of 20 systemic lupus erythematosus (*SLE*) patients with a type I interferon signature (*IFN* ^high^) and 70 IFN^high^ IFNβ-treated multiple sclerosis (*MS*) patients. This confirms our earlier findings of GC-A > GC-B in SLE and GC-B > GC-A in IFNβ-treated MS

In order to validate our observations, publicly available microarray data were downloaded from the Gene Expression Omnibus database of the National Center for Biotechnology Information [32]. Dataset GSE17755 contains gene expression data from peripheral blood cells from 25 healthy individuals and 22 SLE patients [33]. Datasets GSE41846 and GSE41848 consist of combined gene expression data from peripheral blood cells from 38 healthy controls and 78 IFNβ-treated MS patients [34]. Expression data for the 23 IRGs were extracted from these datasets, except for *SAMD9L* as it was not available in all sets. Patients with an IFN signature were selected based on the HC cutoff, as described above, and GC-A/GC-B log-ratios were determined. As shown in Fig. 3, these data confirmed our findings: SLE patients displayed a dominant GC-A score, whereas GC-B dominance was apparent for the majority (78 %) of IFNβ-treated MS patients. A small proportion of IFNβ-treated MS patients had some GC-A dominance, which might be explained by the fact that we could not exclude patients with an IFN signature before start of therapy, as the dataset did not contain paired data before and during IFNβ treatment for all patients. Altogether, these data confirm the presence of IFNα- and IFNβ-specific signatures and the utility of the GC-A/GC-B log-ratio to distinguish between those signatures.

### Expression of gene clusters in other autoimmune diseases

The above results indicate that IFNα and IFNβ-driven type I IFN signatures can be distinguished based on the GC-A/GC-B log-ratio. Thereto, we determined the GC-A/GC-B log-ratio in patients with idiopathic inflammatory myopathies (IIM), RA patients and IFNβ-naïve MS patients, which are autoimmune diseases with type I IFN signatures of yet unknown origin. Again, only IFN^high^ patients were selected.

**Fig. 4:**
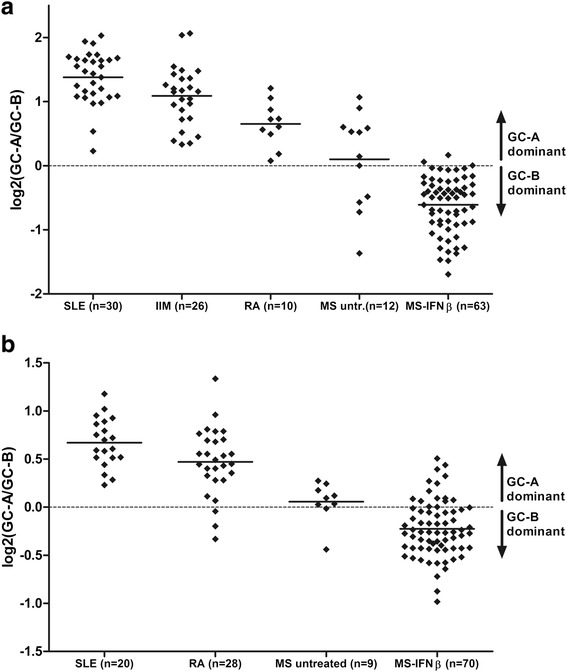
Comparison of gene clusters in autoimmune diseases. **a** Log_2_(GC-A/GC-B) was compared between patients with systemic lupus erythematosus (*SLE*), untreated multiple sclerosis (*MS*) patients and patients with idiopathic inflammatory myopathies (*IIM*) or rheumatoid arthritis (*RA*). The GC-A/GC-B log-ratios are comparable in SLE and IIM. RA patients display less distinctive log-ratios for GC-A and GC-B. Untreated patients with MS are characterized by either GC-A or GC-B dominance. **b** Confirmation of these findings using publicly available microarray data. *IFN* interferon

As shown in Fig. 4a, in patients with IIM there was GC-A dominance, as reflected by the positive GC-A/GC-B log-ratios, indicating predominant IFNα activity similar to that in SLE. The GC-A/GC-B log-ratio in RA patients was lower and approached zero in some of the patients, indicating the contribution of the GC-B cluster as well. Remarkably, untreated MS patients appeared most heterogeneous; approximately half of the patients were characterized by GC-A dominance, whereas the other patients displayed GC-B dominance or a log-ratio close to zero.

These findings were validated for RA patients and untreated patients with MS who were IFN^high^, as the microarray datasets used for validation of our findings in SLE and IFNβ-treated MS patients also contained gene expression data for RA patients (GSE17755, n = 112) and untreated patients with MS (GSE41846 and GSE41848, n = 62) (Fig. 4b) [33, 34].

### Transcriptional regulation of IRG gene clusters

**Fig. 5:**
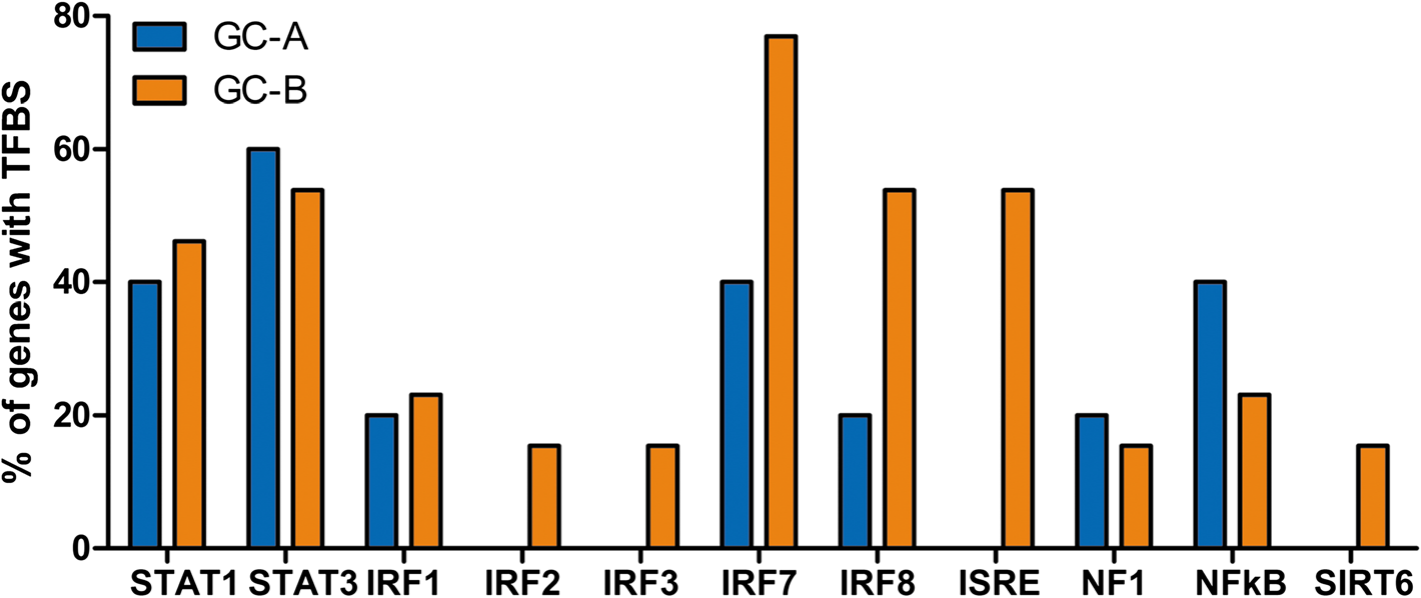
Transcription factor binding site (*TFBS*) analysis using the Interferome database. Represents the presence of transcription factor binding elements 1500 bp upstream from the transcription start site. Interferon regulatory factor (*IRF*)7, IRF8 and interferon-stimulated response element (*ISRE*) are mainly present in genes from GC-B and not in genes from GC-A

To explore functional differences between GC-A and GC-B genes, a transcription factor binding site (TFBS) analysis was performed on these gene clusters using the interferome database and rVISTA [35, 36]. Interestingly, output from the Interferome database showed that prototypical IFN-response elements IFN-stimulated response element (ISRE), interferon consensus sequence-binding protein (ICSBP)/interferon regulatory factor (IRF)8 and IRF7 are mainly present in the GC-B genes and not in the genes of GC-A (Fig. 5). This was supported by statistical analysis of TFBS enrichment, using rVista, which showed significant enrichment in GC-B of both IRF8-binding sites and ISRE (within a 100 bp upstream regulatory region, *p* <0.0001 and *p* = 0.02, respectively). No enrichment of IFN-related TFBS was found in the GC-A gene set (data not shown). This indicates differential transcriptional regulation of the GC-A and GC-B genes, further supporting different upstream activity.

## Discussion

Presence of a type I IFN signature is often discussed as a similarity among autoimmune diseases. In the present study we provide evidence that type I IFN signatures in autoimmune diseases appear less uniform than generally assumed.

IRG expression patterns were different between SLE and IFNβ-treated MS, two autoimmune diseases in which IFN activity has opposing effects on immune pathology and regulation, believed to be a consequence of differential effects of IFNα and IFNβ. Log-ratios of the differentially expressed type I IFN gene clusters, designated GC-A (SLE-IFNα-related) and GC-B (MS-IFNβ-related), revealed excellent separation of patients with SLE and IFNβ-treated MS patients. Moreover, use of the GC-A/GC-B log-ratios in RA, IIM and IFNβ-naïve MS patients provided insight into the origin of the type I IFN signatures in these diseases.

The GC-A/GC-B log-ratios revealed that IIM is predominantly characterized by a GC-A, hence an SLE-like, IFN signature. This fits with the many similarities between SLE and IIM that have been reported previously, including those related to the IFN pathway [37]. With regard to RA, the GC-A/GC-B log-ratio was close to zero for some patients, indicating that both IFNα and IFNβ contribute to the IFN signature, as has been suggested before [38]. Interestingly, GC-A/GC-B log-ratios differed among IFNβ-naïve MS patients, as half of the patients had GC-A dominance, and others had GC-B dominance or ratios close to zero. This could implicate different mechanisms underlying the type I IFN pathway activation in these patients. We previously showed that presence of a baseline IFN signature in patients with MS is related to non-responsiveness to IFNβ treatment [27] and it is highly relevant to further investigate the role of GC-A and GC-B in this perspective, which is the objective of future studies. Altogether, these results suggest a differential role of type I IFNs in autoimmune diseases.

The considerable variance of type I IFNs in humans suggests that, although they bind to the same receptor, the effects they exert might be different. For example, it has been shown that IFNβ is more potent than IFNα in inhibiting proliferation, inducing apoptosis and cell differentiation [39, 40]. Differences were partly explained by the different affinities of type I IFNs for their receptors, resulting in different receptor trafficking, phosphorylation and signaling kinetics [21, 41]. More recently, it has been described that IFNβ can uniquely ligate to IFNAR1, independently of IFNAR2 [42]. Overall, IFNβ appears more potent in activating signal transduction than IFNα, as demonstrated by a more stable receptor complex formation [43], a lower concentration of drug giving the half-maximal response (EC_50_) for ISGF3 phosphorylation [44] and induction of a larger amount of genes than IFNα, especially at long incubation times of 16–36 h [45]. Notably, these long incubation times conform to the chronic IFNα exposure in SLE and 3 months of IFNβ therapy in MS. Interestingly, Moraga et al. hypothesized that the short-term complex formation of IFNα with its receptors might cause a constant low level of apoptosis, whereas the long-term complex formation of IFNβ with its receptors could more potently induce high levels of apoptosis. As SLE pathology is characterized by impaired clearance of apoptotic cells, resulting in immune complex formation and consequent IFNα induction, the low levels of apoptosis as mediated by IFNα could be key to persistence of a vicious pro-inflammatory circle [46]. In MS, however, apoptosis of autoreactive T cells is considered to be one of the anti-inflammatory actions of IFNβ therapy [47].

The implication of IFNα-and IFNβ-specific signatures is supported by the experiments of Der et al., who performed an in vitro experiment in which the fibrosarcoma cell line HT1080 was stimulated with either IFNα or IFNβ, followed by gene expression measurements using oligonucleotide arrays for ±6,800 genes [48]. From these experiments, seven genes overlap with our gene clusters, one from the GC-A cluster and six from the GC-B cluster. *IFITM1*, a GC-A gene, had slightly higher expression in IFNα-stimulated cells compared to IFNβ, whereas the GC-B genes *EIF2AK2*, *IFIT1*, *IFIT*2, *MX1*, *OAS2* and *PLSCR1* were all induced more by IFNβ than by IFNα (1.2-fold to 7-fold higher induction) [48]. Despite the small overlap of genes, the consistency of these results is striking.

It has been suggested that the type I IFN response differs among immune cell types [49], implying that the observed differences between SLE and MS could be partly due to differences in immune cell composition. However, the agreement between our data and those of Der et al. suggests that the observed differences are due to consistent differential signaling in all cells rather than large differences in immune cell compositions or IFNAR expression. However, for replication and complete definition of IFN subtype-specific response programs, whole genome expression studies are required.

Analysis of transcriptional regulation of GC-A or GC-B genes showed enrichment of IRF8/ICSBP binding sites and ISRE in the GC-B cluster. Remarkably, none of the IRGs from the GC-A cluster contained an ISRE, the response element that binds the ISGF3 complex downstream of canonical type I IFN signaling. As they did contain binding sites for STAT1 and/or STAT2, they are probably induced via STAT1-STAT1 monomers or STAT1-STAT2 heterodimers, which both IFNα and IFNβ are able to activate [50, 51]. The observation that these genes are increased in SLE compared to IFNβ-treated MS might be explained by the indication that IFNβ, in contrast to IFNα, might more potently activate a broader range of signaling proteins, including ISGF3 and IRF8, resulting in relatively less activation of the GC-A genes by IFNβ.

Expression of IRF8 is restricted to immune cells and it has the ability to act as a repressor or activator of the IFN response, depending on its interaction partner. A study by Meraro et al. reported that the IRF1-mediated induction of the IFN response gene *ISG15* was inhibited in the presence of IRF8, whereas interaction of IRF8 and PU.1 synergistically enhanced *ISG15* induction [52]. This suggests an immunomodulatory role for IRF8, which might be key to the different effects of IFNα and IFNβ on the immune system.

## Conclusions

Conclusively, this study demonstrated that the IFN signatures display distinct differences between autoimmune diseases. Considering the pro-inflammatory nature of IFNα in SLE and the anti-inflammatory role of IFNβ in MS, specification of the type I IFN response in autoimmune diseases might give new insights into its role in disease pathology and/or its therapeutic potential.

## Additional file

Additional file 1: Table S1. List of 23 type I interferon (IFN) response genes that were measured. **Table S2.** List of genes that are significantly differentially expressed between patients with systemic lupus erythematosus (SLE) and patients with multiple sclerosis (MS) who were treated with IFNβ. (PDF 116 kb)

## Abbreviations

bp: base pair(s)
GAS: interferon-gamma activated sequence
HC: healthy control
ICSBP: interferon consensus sequence-binding protein
IFN: interferon
IFN^high^: presence of a type I interferon signature
IIM: idiopathic inflammatory myopathies
IL: interleukin
IL-1R: interleuking-1 receptor
IRF: interferon regulatory factor
IRG: interferon response gene
ISGF: interferon-stimulated gene factor
ISRE: interferon-stimulated response element
MS: multiple sclerosis
RA: rheumatoid arthritis
SLE: systemic lupus erythematosus
TFBS: transcription factor binding site
TNF: tumor necrosis factor
