# Supplementary material for: Physiological evidence for diversification of IFNα- and IFNβ-mediated response programs in different autoimmune diseases
Source: Arthritis Res Ther. 2016 Feb 17;18:49. doi: 10.1186/s13075-016-0946-9 (PMC4756531; doi:10.1186/s13075-016-0946-9)
Supplement: Additional file 1: Table S1. — List of 23 type I interferon (IFN) response genes that were measured. Table S2. List of genes that are significantly differentially expressed between patients with systemic lupus erythematosus (SLE) and patients with multiple sclerosis (MS) who were treated with IFNβ. (PDF 116 kb) [file 13075_2016_946_MOESM1_ESM.pdf]

Table S1: List of 23 type I IFN response genes that were measured

Gene Symbol	Gene Name
EIF2AK2	eukaryotic translation initiation factor 2-alpha kinase 2
EPSTI1	epithelial stromal interaction 1
HERC5	HECT and RLD domain containing E3 ubiquitin protein ligase 5
IFI27	interferon, alpha-inducible protein 27
IFI44L	interferon-induced protein 44-like
IFI6	interferon, alpha-inducible protein 6
IFIT1	interferon-induced protein with tetratricopeptide repeats 1
IFIT2	interferon-induced protein with tetratricopeptide repeats 2
IFITM1	interferon induced transmembrane protein 1
IFITM3	interferon induced transmembrane protein 3
IL1RN	interleukin 1 receptor antagonist
LGALS3BP	lectin, galactoside-binding, soluble, 3 binding protein
LY6E	lymphocyte antigen 6 complex, locus E
MX1	MX dynamin-like GTPase 1
OAS2	2'-5'-oligoadenylate synthetase 2, 69/71kDa
OAS3	2'-5'-oligoadenylate synthetase 3, 100kDa
PARP12	poly (ADP-ribose) polymerase family, member 12
PLSCR1	phospholipid scramblase 1
RSAD2	radical S-adenosyl methionine domain containing 2
RTP4	receptor (chemosensory) transporter protein 4
SAMD9L	sterile alpha motif domain containing 9-like
TNFAIP6	tumor necrosis factor, alpha-induced protein 6
TNFSF10	tumor necrosis factor (ligand) superfamily, member 10

Table S2: Genes differentially expressed between SLE and IFNβ-treated MS patients

Gene name	Fold difference	Adjusted P value
Upregulated in SLE patients (GC-A)		
**IFI27**	7.56	<0.001
**IFI44L**	1.39	0.006
**IFITM1**	1.69	<0.001
**LY6E**	1.32	0.006
**TNFAIP6**	1.73	0.003
Upregulated in IFNβ-treated MS patients (GC-B)		
**EIF2AK2**	1.75	<0.001
**HERC5**	4.63	<0.001
**IFIT1**	3.11	<0.001
**IFIT2**	3.86	<0.001
**IL1RN**	3.11	<0.001
**LGALS3BP**	1.68	<0.001
**MX1**	1.14	0.044
**OAS2**	1.77	<0.001
**PARP12**	1.27	0.009
**PLSCR1**	1.59	<0.001
**RSAD2**	1.93	<0.001
**SAMD9L**	1.92	<0.001
**TNFSF10**	2.13	<0.001
